# Supplementary material for: TGF-β Induced by Allergic Lung Inflammation Enhances Os-Teosarcoma Lung Metastasis in a Mouse Comorbidity Model
Source: Int J Mol Sci. 2025 May 24;26(11):5073. doi: 10.3390/ijms26115073 (PMC12155057; doi:10.3390/ijms26115073)
Supplement: Supplementary file 1 [file ijms-26-05073-s001.zip › ijms-3559436-supplementary.pdf]

Supplementary Table S1.

The input parameters used to find out the percentage of tissue stained with the immunohistochemical marker.

Positive pixel count algorithm input parameters

**Total Pixel**	
Colour Saturation Threshold	0.04
Iwp (High)	220
Iwp (Low) = Ip (High)	175
Ip (Low) = Isp (High)	100
Isp (Low)	0
Inp (High)	-1
Nuclear Pixel	
Min Nuclear Size (um^2)	10
Max Nuclear Size (um^2)	1000
Min Roundness	0.1
Min Compactness	0
Min Elongation	0.1
Remove Light Objets	0
Weak (1+) Threshold	210
Moderate (2+) Threshold	188
Strong (3+) Threshold	162

The input parameters used to find out the percentage of tissue stained with the immunohistochemical marker.

Supplementary Figure S1

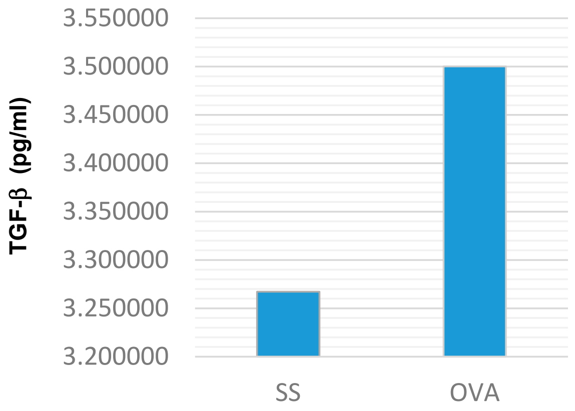

Supplementary Figure S1. Quantification of TGF-β levels by Cytometric Bead Array (CBA) in serum samples. BALB/c mice were treated with ovalbumin (OVA) to induce allergic airway inflammation or received saline solution (SS) as control. TGF-β levels were significantly increased in the OVA-treated group compared to SS controls.
